# A Global Call for More Investment in Data-Driven Perioperative Care Delivery Models in Humanitarian Settings

**DOI:** 10.1007/s00268-023-06962-1

**Published:** 2023-03-06

**Authors:** Busisiwe Mrara, Olanrewaju Oladimeji

**Affiliations:** 1 Anaesthesiology and Critical Care, Faculty of Health Sciences, Walter Sisulu University, Mthatha, 5099 South Africa; 2 Department of Public Health, Faculty of Health Sciences, Walter Sisulu University, Mthatha, 5099 South Africa

## Introduction

In 2015, the Lancet commission on global surgery reported a lack of access to safe, lifesaving, and affordable surgery affecting up to five billion people globally, with 90% living in low- and middle-income countries (LMIC) [1]. This deficiency is due to a lack of medical expertise, equipment, and infrastructure. Despite widespread global surgery advocacy, natural disasters, war, and conflict have slowed progress towards strengthening health systems to address this shortage. Humanitarian surgical missions are emerging as a critical solution for providing much-needed surgical care to the world's impoverished nations. According to a survey of surgical non-governmental organizations, the annual case load is 2.9 million. This figure is even presumed to be underreported [2]. These missions have served a variety of purposes, including the treatment of civilian traumatic injuries. Other aspirations include bringing complex surgeries such as cardiac and craniofacial surgery to underserved areas and providing critical surgical procedures to underserved health systems. As a matter of fact, humanitarian surgical work is gaining prominence, fostering mutually beneficial collaboration between high income country (HIC) and LMIC health systems while also providing surgical experiential training opportunities. The expansion of the scope and responsibilities of humanitarian work necessitates greater attention to detail, monitoring, and governance to ensure quality and a consistent positive impact on surgical workload. This is easier said than done because working conditions are frequently unfavorable due to a lack of resources and environmental issues affecting logistics and data management. A few well-established surgical non-governmental organizations (NGOs) (such as Operation Smile) regularly report their outcomes. These results are comparable to HIC settings and are improving [3]. However, much of the surgical work done in humanitarian settings goes unreported in terms of quality indicators and medium and long-term outcomes. As a result, little attention has been paid to the impact of rudimentary unstandardized perioperative assessment systems on outcomes in humanitarian surgery.

Patients in humanitarian settings face unique perioperative challenges, such as advanced pathology from delayed access to care, the unquantifiable impact of local traditional therapies, HIV, anemia, and malnutrition. Despite younger age and fewer comorbidities, the African surgical outcomes study discovered twice the perioperative mortality in the African cohort compared to the global average [4], indicating the need for further investigation. The logistics of timely consultations in remote rural areas, the language barrier, and the cost and availability of appropriate investigations are all barriers to preoperative evaluation in these settings. Assessing patients prior to anesthesia and surgery has been shown to improve outcomes by identifying potential anesthetic difficulties, allowing for medical condition optimization, planning postoperative care, and finally, assessing and quantifying perioperative risk using a scoring system. The scoring system quantifies risk and assists with patient counselling, promoting informed consent and patient-centered outcomes. Various perioperative risk scoring systems that incorporate patient and surgical factors have been used to aid in the assessment. However, some pitfalls to using them have been identified in the literature [5]. Most risk scores, for example, are developed for specific target populations and types of surgery and, as a result, have been found to be less predictive and have limited applicability in all settings. Furthermore, some of the more recently developed tools compute a score by combining multiple variables and information from chronic comorbidities, blood test results, and previous contact with the health system. In humanitarian settings, information is frequently unavailable. Furthermore, the cost, resources, and time investment in developing, validating, and testing risk predictive models is prohibitively expensive, necessitating the collection of big data through electronic health records, fast internet, data management and storage, and time dedicated to patient care. These resources are inaccessible during humanitarian crises.

Wild and colleagues carried out a scoping review of the literature on "Perioperative Risk Assessment in Humanitarian Settings" for this issue of WJS [6]. Due to the scarcity of records, the authors searched traditional data bases, including all study designs, with no restrictions on search languages. They were able to find and analyze only 50 full-text articles containing data from over 37 countries, mostly in Africa, the Middle East, and Asia. They were unable to locate any perioperative risk assessment models created for humanitarian surgery. Models developed from data in LMICs, on the other hand, have limited applicability in humanitarian settings because they require information such as comorbidities, which is not always available. Another issue is the usability of models that rely on online calculations. In addition, they highlighted the inconsistency of HIC-derived models in humanitarian settings. Their findings are significant because they explain the scarcity of reliable records that can be used to build perioperative risk models. Due to unregulated event documentation, they discovered inconsistencies in the collection of critical data such as preoperative clinical status, surgical indications, and postoperative outcomes. This is because unstructured paper-based recording systems are used, as well as the possibility of prioritizing patient care over data collection. Their findings support the need for better governance and monitoring of humanitarian surgery. Significant investment in perioperative registries, as well as standardized data collection methods, will be required. This will enable the development of tailored perioperative risk prediction models by facilitating the creation of pragmatic minimal datasets that include quality indicators such as short and long-term outcomes. Among the resources that can be budgeted for in humanitarian missions are electronic health records, high-speed internet, and dedicated data management expertise. Tracking perioperative mortality in all countries as a foundation for quality improvement is a key focus of the Global Surgery 2030 plan, and quality data will aid in this endeavor.
